# Pediatric Inflammatory Myofibroblastic Tumors of the Airway: Two Case Reports with Varying Clinical Presentation

**Published:** 2018-05

**Authors:** Nuthan Kumar, Thirunavukkarasu Saravanamuthu, Arathi Srinivasan, Thulasi Raman, Julius-Xavier Scott

**Affiliations:** 1 *Department of Pediatric Hematology and Oncology, Kanchi Kamakoti Childs Trust Hospital And The Childs Trust Medical Research Foundation, Nungambakkam, Chennai – 600034,Tamil Nadu State, India.*; 2 *Department of Pediatric ENT, Kanchi Kamakoti Childs Trust Hospital And The Childs Trust Medical Research Foundation, Nungambakkam, Chennai – 600034,Tamil Nadu State, India.*; 3 *Department of Pathology, Kanchi Kamakoti Childs Trust Hospital And The Childs Trust Medical Research Foundation, Nungambakkam, Chennai – 600034,Tamil Nadu State, India. *

**Keywords:** Airway, Children, Inflammatory, Myofibroblastic, Recurrence

## Abstract

**Introduction:**

An inflammatory myofibroblastic tumor (IMT) is a rare tumor of intermediate malignant potential. It may occur in a wide range of anatomical locations. One-third are found in the respiratory tract. We report two cases of IMT of the airway diagnosed at our institution.

**Case Report:**

Case 1: A 6-year-old male child presented with a 1-month history of hoarseness of the voice. On evaluation, a polypoid nodule was noted in the right vocal cord which was excised through the endolaryngeal route. Histopathology was suggestive of anaplastic lymphoma kinase (ALK)-negative IMT. He presented with recurrence after 4 months, for which he underwent endolaryngeal reexcision and tracheostomy for airway protection. A third recurrence after 6 months was managed with laser excision, and the patient was started on oral celecoxib. After 1.5 years of follow up, endoscopic examination showed no recurrence, and celecoxib was continued. Case 2: A 7-year-old male child presented with cough and respiratory distress. Bronchoscopy and high resolution computed tomography showed a polypoidal lesion with calcification arising from the left anterolateral wall of the trachea with significant narrowing of the lumen. The patient underwent biopsy followed by endoscopic excision, and was diagnosed with IMT. Currently the patient is under follow up with no recurrence.

**Conclusion:**

IMT indicates a proliferative myofibroblastic growth. Surgical resection should be recommended for all lesions if not prohibited by anatomic location or morbidity. Patients should be followed up closely for recurrence. In most cases, complete surgical excision will suffice; however multiple recurrences can be managed with chemotherapy. These two cases highlight the importance of a multidisciplinary approach in rare tumors in difficult anatomical locations.

## Introduction

An inflammatory myofibroblastic tumor (IMT) is a rare tumor with an intermediate malignant potential occurring mainly in children and young adults (1,2). These tumors rarely metastasize, but frequently recur (3). Histopathologically, the tumors consist of various inflammatory cells ranging from primarily myofibroblastic to a heavy infiltration of plasma cells (4). IMT is known by numerous terms, including plasma cell granulomas, inflammatory pseudotumor, fibrous histiocytoma and pseudolymphoma (5), and may occur in a wide range of anatomical locations such as the lungs, omentum, bladder, spleen, breast, pancreas, liver, colon, spermatic cord, prostate, peripheral nerves, soft tissue, and orbit. About one-third of these tumors are found in the respiratory tract (6). We report two cases of IMT of the respiratory tract; one in the larynx and the other in the trachea.

## Case Report

*Case* *1: *A 6-year-old boy who was previously well presented to us with a history of hoarseness of the voice over the past 1 month and breathing difficulties for the previous 2 weeks. There was no history of trauma, or any previous surgery of the head and neck region except for a tongue-tie release during the infantile period. There was no history of fever or loss of weight or appetite.

**Fig 1:**
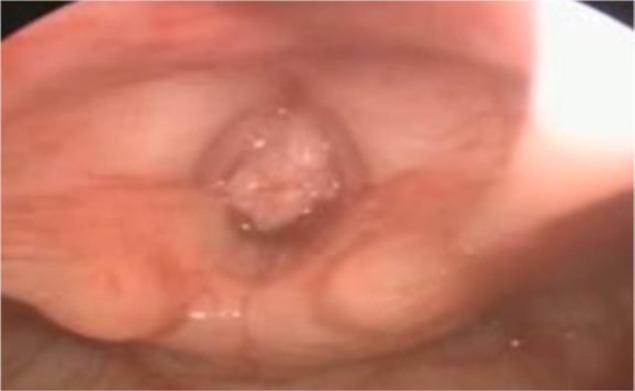
Laryngoscopy showing a mass in the free margin of the cords

On examination, the patient’s vital signs were stable. Systemic examination did not reveal any abnormality. Flexible laryngoscopy showed a polypoidal mass approximately 1 × 1 cm in size in the free border and the undersurface of the right vocal cord extending down to the subglottis (Fig.1).

**Fig 2:**
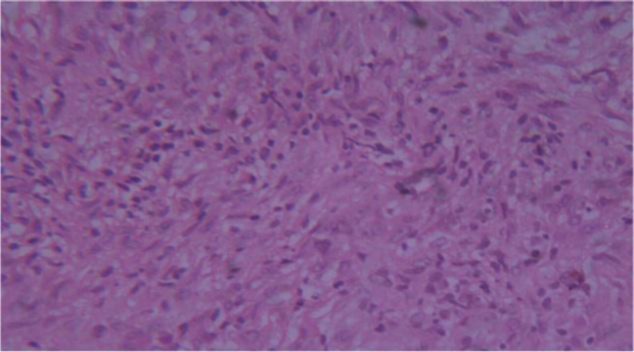
H&E 40× showing spindle-cell proliferation against background of inflammatory cells

The patient was evaluated with computed tomography (CT) of the neck, which revealed a polypoid nodule of size 1 × 1 cm in the right vocal cord projecting into the lumen with no evidence of extension into the anterior commissure or deep cartilage. An initial impression of papilloma was made. The patient underwent microlaryngeal gross total excision of the mass under general anesthesia. Histopathology of the excised mass showed polypoid fragments of the respiratory epithelium and metaplastic squamous epithelium. The subepithelium showed spindle-cell proliferation with storiform appearance and tiny microhemorrhages. There were dense infiltrates of lymphocytes, plasma cells, and eosinophils. The spindle cells did not show nuclear atypia or mitosis and possessed vesicular nuclei with conspicuous nucleoli (Fig.2).

**Fig 3:**
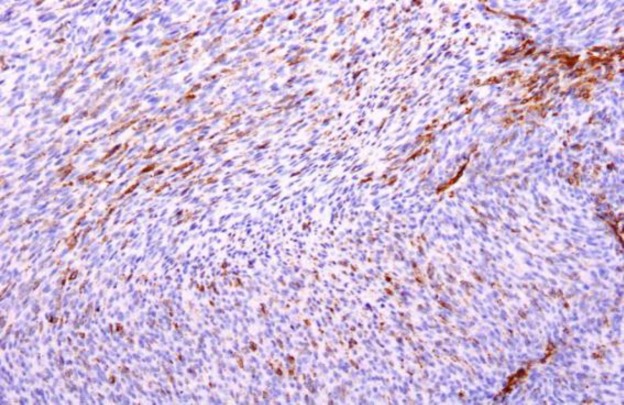
Tumor cells showing positivity for smooth muscle actin on immunohistochemistry

 Immunohistochemistry was positive for vimentin and smooth muscle actin (SMA) (Fig.3), negative for cytokeratin, desmin, anaplastic lymphoma kinase (ALK), S100 and cluster of differentiation 34 (CD34). These features were thus suggestive of a spindle-cell neoplasm favoring IMT.

The patient was followed up without any adjuvant therapy. After 4 months, he presented with repeat symptoms of hoarseness of voice. Laryngoscopy revealed a recurrent mass, 2 × 2 cm in size. He underwent a microlaryngeal excision using microdebrider, and also required a tracheostomy for airway protection. The post-surgery period was uneventful, and the patient was placed on regular tracheostomy care. After a 6 months of an uneventful follow-up period, a routine laryngoscopic examination showed a recurrent mass of 1 × 1 cm, and an endolaryngeal diode laser excision of the mass was performed. In view of the recurrent nature of the mass, the patient was started on adjuvant oral celecoxib at a dose of 100 mg/m^2^/day. The tracheostomy was decannulated successfully after 6 months. Currently, 1.5 years after the last surgery, the patient is healthy with no evidence of any recurrence, and is being continued on celecoxib therapy.

*Case 2:*

A 7-year-old male child presented with cough and respiratory distress of 15 days duration. There was no history of fever, previous history suggestive of bronchial asthma, nor any foreign body inhalation. On examination, the patient was tachypneic. On auscultation, there was mild stridor, but other systemic examination was normal. A trial of bronchodilators and antibiotics was made, but as the symptoms persisted, he was evaluated further. Bronchoscopy and high resolution CT showed a polypoidal lesion with calcification arising from the anterolateral wall of the lower trachea with significant narrowing of the lumen. The patient underwent biopsy followed by endoscopic laser excision, and histology was suggestive of IMT. Currently the patient is under follow up without any adjuvant therapy and has had no recurrence so far.

## Discussion

IMT is one of the rare low-to-intermediate grade sarcomas. Initially it was thought to be an inflammatory response to various stimuli, but recent studies have proved IMT to be neoplastic and can recur locally and metastasize (7). It has also been suggested that trauma, surgery, autoimmune etiologies, inflammation, and infections such as Epstein-Barr virus or human herpes virus could result in the development of IMT (8).

IMT was first described in the lungs but later was also found in other sites such as the orbit, spleen, genitourinary tract, mesentery, cardioesophageal junction, breast, central nervous system, and larynx. The larynx has been a very rare site for involvement in IMT (9). Wenig et al. reported the first case series on IMT in the larynx in 1995, which included eight cases ranging from 19–69 years in age (10). Alhumaid et al. reviewed all the laryngeal IMT data published in the literature, which included a total of 31 cases from various parts of the larynx involving varied age groups (11). IMT of the larynx in the pediatric age up has been reported in only a few case series, with the youngest patient being a 19-month old child reported by Das Purkayastha et al. (12).

IMTs have been described in various parts of the larynx, with the true vocal cords being the most common site, although it is also described in the subglottis and supraglottis regions. In a review by Alhumaid et al., 19 cases were described in the glottis region followed by seven in the subglottis, three in the supraglottis and two in other regions (11). The regions involved in our index cases were the right-sided vocal cord and the left anterolateral wall of the trachea. No gender predilection is described in the literature.

The clinical presentation of these tumors depends on the site of origin of the mass. IMT in the laryngeal region usually presents with multiple symptoms. Voice change is the most common symptom, followed by stridor, dyspnea, cough, apnea, and respiratory failure (11). A nonspecific syndrome of chronic malaise, fever, and weight loss associated with raised inflammatory markers is also a recognized presentation (6). Hoarseness of the voice and respiratory distress with cough were the presenting complaints in our cases.

Investigations such as chest X-ray, CT scan of the neck with contrast, and laryngo- bronchoscopy with biopsy for histology and immunohistochemical staining are essential in the diagnosis of these tumors. Histopathology features which help in the diagnosis of IMT include the absence of necrosis, absence of atypical figures of mitosis, a mitotic rate of less than two percent/10 HPF, mild cellular pleomorphism, absence of infiltrating growth, and presence of polyclonality of plasma and mixed inflammatory infiltrate. Ultrastructure studies may show spindle cells with elongated cytoplasmic processes. The immunocytochemistry positivity for vimentin and SMA with morphological features and negativity for cytokeratin and CD34 help to confirm the diagnosis of IMT (13). A specific clonal cytogenetic rearrangement has been found in approximately 30% of children with IMT that activates the ALK receptor tyrosine kinase gene on chromosome band 2p23 (3).

Many differential diagnoses have been described for IMT, such as epidermoid carcinomas with spindle-shaped cells,malignant mesenchymatous tumors (fibrosarcomas, chondrosarcomas, histiocytomas and others) and lymphomas. True IMT must be distinguished from inflammatory pseudotumors developing in response to a healing process, injury, or infection.

Standard therapy of IMT is always surgical excision whenever possible (4). The treatment of respiratory tract IMT is also primarily surgical (6). Spontaneous resolution has been reported in a very few asymptomatic cases (14). Prognosis of IMT is related to tumor size, histopathology, and completeness of the surgical excision. Significant mortality and morbidity have been reported for attempted surgical excision of tumors in challenging anatomic areas. Recurrences are known to be more frequent if complete excision is not performed due to technical difficulties such as anatomical site or the multinodular nature of the lesion.

Endoscopic excision with or without steroids is considered to be first-line treatment for laryngeal IMTs, as only few recurrences have been reported in literature (15). Open excision is usually advocated in cases of recurrence, poor endoscopic visualization, or when malignancy cannot be excluded. The recurrence rate of laryngeal IMT is approximately 18% (2). The review by Alhumaid et al. also suggested the management of this disease was best achieved by microlaryngoscopies (with or without laser) and complete excision with or without steroid therapy. The recurrent cases were managed with surgery combined with chemotherapy (11). Definitive therapy for recurrent tumors can also be achieved by local laser therapy. In a case series by Brodlie et al., residual tumor was removed by a laser technique twice and the patient remained well clinically over the following 18 months (6). 

One of our index cases underwent microlaryngeal complete excision without any recurrence. However, the other child underwent microlaryngeal gross total excision initially followed by removal using a microdebrider. At second recurrence, a laser was used for endolaryngeal complete excision, supplemented with celecoxib.

In cases where surgical resection is not possible, or with recurrent disease after resection, additional treatment options such as chemotherapy are needed. Several different regimens have been described for recurrent disease, with cyclophosphamide, cyclosporine, methotrexate, corticosteroids and non-steroidal anti-inflammatory drugs (NSAIDs) (4,16). The use of anti-inflammatory therapy for IMT was first proposed by Hakozaki et al. based on their understanding of the pathophysiology of the disease (17). Johnson et al. treated a case of pulmonary IMT with a chemotherapy regimen consisting of vincristine, ifosfamide, and doxorubicin followed by celecoxib for 3.5 years after completion of the therapy, and the patient has a complete response with regression of all identifiable disease and has been disease-free for more than 4 years. The same authors have also successfully treated another patient with a hepatic IMT with non-steroidal anti-inflammatory drug therapy. Complete resolution of the tumor was achieved within 1 year of initiating the treatment (4). In a series by Mehta et al., one patient with a primary leg tumor developed lung metastases, and is a long-term survivor with chemotherapy alone consisting of ibuprofen, cyclophosphamide, doxorubicin and vinblastine, even though the primary tumor and metastatic lesions were unresectable (3). Another 14-year-old girl reported by Tao et al. with intrabdominal IMT was treated with a chemotherapy regimen consisting of six courses of methotrexate (20 mg/m^2^) and cisplatin (30 mg/m^2^) administered once a month, associated with oral administration of slow-release diclofenac sodium until cessation of chemotherapy. Currently, the patient is alive without recurrence after 3 years of follow up (18). We have used oral celecoxib at a dosage of 100 mg/m^2^/day in one of our cases due to the recurrent nature of the tumor without any recurrence. The exact mechanism behind the effectiveness of anti-inflammatory therapy in the treatment of IMT is not known. It is postulated to be due to blocking of the mediators of angiogenesis, including vascular endothelial growth factor, specifically by cyclo-oxygenase-2 (COX 2) inhibitors (19).

Crizotinib, a molecule inhibitor of ALK, has shown promising results in adults with ALK-positive IMT (20). In ALK^+^ tumors, the ALK gene on chromosome 2 is fused to one of several partners, such as non-muscular tropomyosin 3-ALK (TPM3-ALK), which can confer proliferative and metastatic properties to the tumor (21). However, there is not much evidence for its use in the pediatric population. The role of radiotherapy is not clear, but in a few cases it has been used for recurrent cases (2).

## Conclusion

IMTs are rare tumors, and involvement of the larynx is still rarer in the pediatric population. Thorough investigation and access to a multidisciplinary team are essential in making a definitive management plan. The mainstay of therapy should continue to be complete endolaryngeal excision with or without laser treatment. In cases where surgical resection is not possible, or with recurrent disease after resection, chemotherapy options are needed. Owing to its rarity, there are no definite chemotherapeutic guidelines, and various drugs have been tried. NSAIDS are a new feasible therapeutic choice. However, further research into anti-inflammatory therapies and their mechanism and ideal duration of therapy may improve our understanding and treatment of IMT.
